# Foliar pathogen epidemic slows decomposition of invasive plant litter

**DOI:** 10.1002/ecy.70374

**Published:** 2026-04-09

**Authors:** Brett R. Lane, Chris Wojan, Carlie Meehan, Amy E. Kendig, Robert D. Holt, Philip F. Harmon, Keith Clay, S. Luke Flory, Erica M. Goss

**Affiliations:** ^1^ Department of Plant Pathology University of Florida Gainesville Florida USA; ^2^ Department of Plant Pathology University of Minnesota St. Paul Minnesota USA; ^3^ Department of Agronomy Kansas State University Manhattan Kansas USA; ^4^ Department of Biology Indiana University Bloomington Indiana USA; ^5^ Agronomy Department University of Florida Gainesville Florida USA; ^6^ Department of Biology University of Florida Gainesville Florida USA; ^7^ Ecology and Evolutionary Biology Tulane University New Orleans Louisiana USA; ^8^ Invasion Science Institute, University of Florida Gainesville Florida USA; ^9^ Emerging Pathogens Institute, University of Florida Gainesville Florida USA

**Keywords:** *Bipolaris gigantea*, decomposition, lignin, microbial communities, *Microstegium vimineum*, stiltgrass

## Abstract

Decomposition of plant litter, facilitated primarily by microbial decomposers, plays a critical role in biogeochemical cycling and ecosystem function. The rate of litter decomposition can determine its environmental impact, where accelerated decomposition alters the timing and rate of nutrient release and may promote nutrient leaching, whereas slowed decomposition can result in litter accumulation, which impacts seedling recruitment, fire regimes, perennation of microbial communities, and slows nutrient release. Mutualistic endophytes are known to slow litter decomposition, but less is known about the impact that plant pathogens, present in diseased litter, have on decomposition rates. We compared litter decomposition of the invasive annual grass *Microstegium vimineum* with Bipolaris leaf spot symptoms, a fungal disease, to litter without symptoms of the disease in a year‐long common garden experiment. We found leaf tissue with disease symptoms decomposed later in the year compared to litter without symptoms. By summer, 54% of leaf tissue from healthy sites remained compared to 80% of leaf material from diseased litter. Fungal infection did not impact the lignin or C:N content of the litter. There were significant differences in fungal community composition between infected and healthy litter at the start of the experiment that persisted until the end of summer. Disease epidemics prior to senescence contributed to the persistence of infected tissue, which could slow the return of nutrients to the environmental pool and promote the survival and dispersal of pathogen inoculum the following season.

## INTRODUCTION

Plant litter decomposition is a critical component of ecosystem function, with numerous environmental impacts, including mineralization of organic nutrients, generation of soil organic matter, and return of CO_2_ fixed during photosynthesis to the atmosphere (Canessa et al., 2021; Vivanco & Austin, 2019). The timing and rate of litter decomposition contribute to its environmental impact, where accelerated decomposition may promote nutrient leaching, while slowed decomposition results in litter accumulation, with impacts on seedling recruitment, fire regimes, and perennation of microbial communities (Dubeux & Sollenberger, 2020; Facelli & Pickett, 1991). Microbes are responsible for the vast majority of the decomposition of terrestrial plant litter (Berg & McClaugherty, 2003), but the rate of litter decomposition can be significantly impacted by the phyllosphere community, including mutualistic endophytes, prior to host senescence (Purahong & Hyde, 2011). Mutualistic endophytes have been shown to drive alterations to the chemical make‐up of the plant, specifically through the production of toxic alkaloids, resulting in slowed decomposition post litter‐fall (Lemons et al., 2005; Omacini et al., 2004; Purahong & Hyde, 2011). In contrast, phytopathological research has focused more on monitoring the survival of pathogen inoculum in litter, rather than the effects of pathogens on the rates or mechanisms of litter decomposition. As climate change promotes the emergence and spread of plant pathogens (Bebber, 2015), it is important to understand the role that pathogens play in decomposition dynamics.

Pathogen colonization of host material prior to senescence may alter the chemical composition through multiple potential mechanisms, including secretion of cell wall degrading enzymes, detoxification of antimicrobial plant secondary metabolites, and induction of host defense responses prior to senescence (Dangl & Jones, 2001; Kubicek et al., 2014). These molecular processes subsequently could speed or slow decomposition. Alternatively, changes to plant‐associated fungal communities during disease epidemics could affect the prevalence of fungi that serve key roles in litter decomposition by breaking down complex macromolecules (Purahong et al., 2016).

**FIGURE 1:**
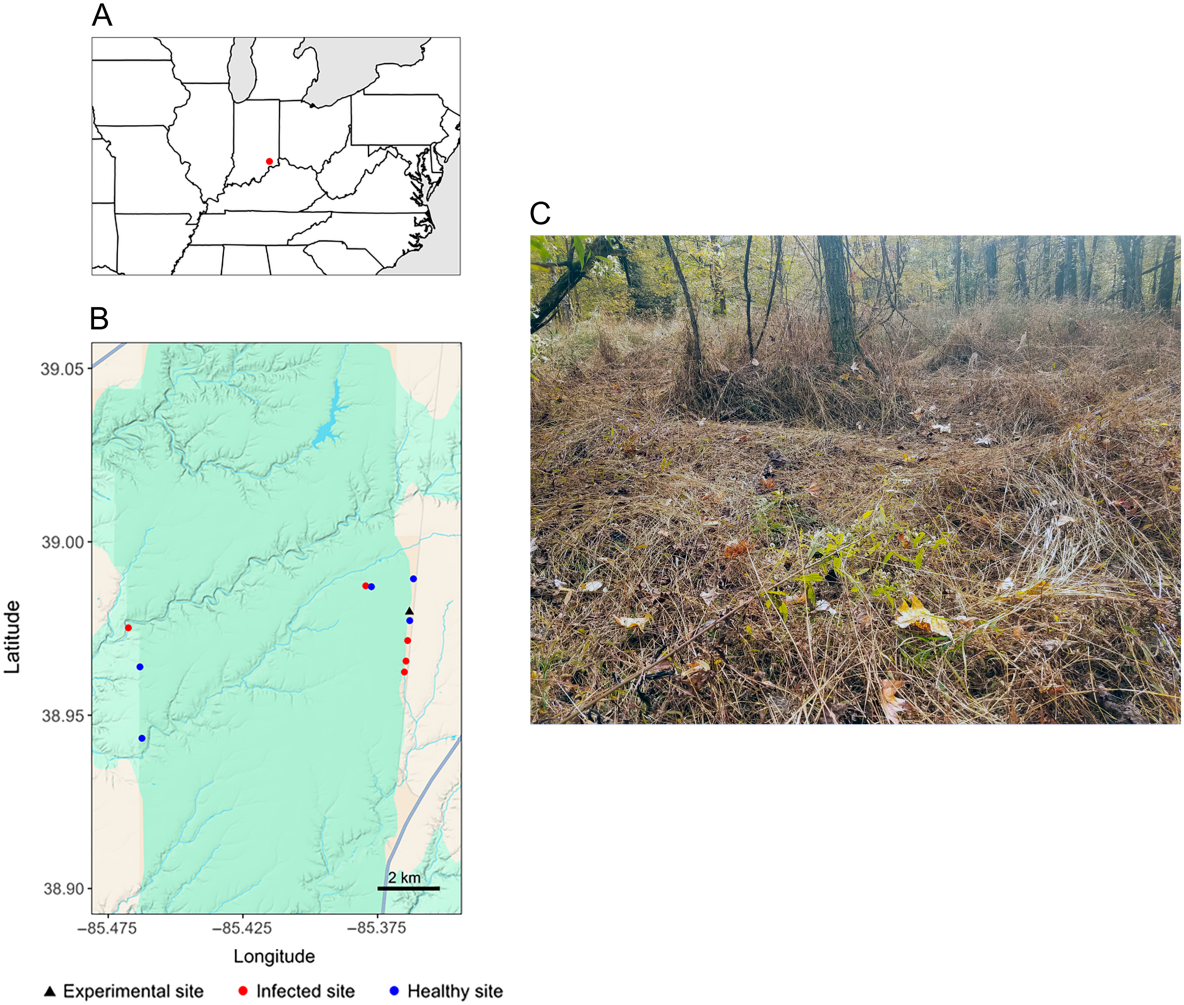
(A) Location of Big Oaks National Wildlife Refuge in southeastern Indiana, USA. (B) Map of litter collection sites within the refuge (blue dots indicate healthy sites and red dots indicate infected sites). The black dot indicates the experimental site; See also Appendix S1: Table S1. Collection sites were clustered around the borders of the wildlife refuge due to access limitations (i.e., it is a former military testing facility). (C) Naturally senesced *M. vimineum* litter blanketing infected site no. 3. Photo was taken by Brett R. Lane.

Here, we investigate the impact of an emerging foliar pathogen on plant litter decomposition dynamics. The study host is *Microstegium vimineum*, a C4 annual grass, native to Asia, that has widely invaded the eastern United States. Impacts of *M. vimineum* on native plant communities are caused, in part, by accumulation of thick layers of slowly decomposing litter that inhibit recruitment and growth of native species (Ehrenfeld et al., 2001; Flory & Clay, 2010) (e.g., Figure 1C). Flory et al. (2011) first reported a leaf spot disease epidemic on *M. vimineum* at Big Oaks National Wildlife Refuge (BONWR) in southeastern Indiana, USA. Subsequent studies have established that annual disease epidemics occur on *M. vimineum* at BONWR and throughout the central part of its invaded range, and that they are caused primarily by the fungus *Bipolaris gigantea* (Lane et al., 2020, 2023; Stricker et al., 2016). The patchy distribution of disease on *M. vimineum* within BONWR creates an ideal study system to investigate foliar pathogen impacts on litter decomposition. We used the *M. vimineum–B. gigantea* system as a model to test the hypotheses that (1) senescent litter consisting of diseased plant material colonized by *B. gigantea* decomposes differently than *M. vimineum* litter free of Bipolaris leaf spot symptoms, and (2) differences in decomposition between infected and healthy litter are associated with altered litter chemistry and different microbial assemblages during litter decomposition. The experiment was conducted in a common garden to account for the possibility of altered decomposition dynamics following translocation; thus, we also used comparisons between healthy translocated and healthy non‐translocated litter to ask if *M. vimineum*, like native species, experiences more rapid decomposition at the site of origin, known as “home‐field advantage” (Ayres et al., 2009).

## MATERIALS AND METHODS

### Site description

BONWR in southeastern Indiana, USA, located 850–900 feet above sea level, is a 50,000‐acre wildlife refuge comprised of approximately 27,000 acres of upland forests. These temperate deciduous forests contain both evergreen and deciduous species. Our experimental common garden site was primarily dominated by deciduous red maple (*Acer rubrum*) and sweetgum (*Liquidambar styraciflua*). In the early 1900s, the land was used for farming and grazing before being purchased by the United States Army in 1940 for munitions testing and was allowed to rewild. In 2000, the land was turned over to the United States Fish and Wildlife Service. Public access to the wildlife refuge is limited. The hottest month is July with an average high of 31.1°C, the coldest month is January with an average low of −4.6°C. The mean annual precipitation is 1295 mm (NOAA/NCEI, 2021; USFWS, 2000).

### Site selection and litter preparation

*Microstegium vimineum* litter used in this experiment was collected from discrete sites of Bipolaris leaf spot disease and from sites not known to have symptoms of Bipolaris leaf spot disease (Benitez et al., 2022; Kendig et al., 2022; Lane et al., 2020, 2023). Bipolaris leaf spot symptoms (or lack of) were confirmed through scouting efforts in the summer months prior to litter collection and by inspection for signs of *B. gigantea* as described below. We henceforth refer to litter collected from sites with signs and symptoms of Bipolaris leaf spot as “infected litter” and litter free of signs and symptoms of Bipolaris leaf spot as “healthy litter.” Following natural senescence, we measured decomposition at a single, independent, healthy site (henceforth common garden site).

On September 23, 2019, prior to senescence, we collected 20 leaves from each site to confirm the presence or absence of *B. gigantea*. At sites with zonate eyespot lesions (see pictures Lane et al., 2020) on *M. vimineum*, confirmation that symptoms were caused by Bipolaris leaf spot was achieved by placing the collected symptomatic leaves in a plastic bag with a damp paper towel for 48 h to induce sporulation of *B. gigantea* conidia which were identified by their unique morphology and size using a stereoscope. This procedure was repeated with randomly selected leaves from healthy sites (as none contained eyespot lesions) to confirm *B. gigantea* was not observed.

We collected naturally senesced *M. vimineum* litter during October 26–November 1, 2019, from five infected, five healthy, and the healthy common garden site at BONWR, a temperate deciduous forest (Figure 1; Appendix S1: Table S1). At all sites, invasive *Microstegium* litter was conspicuously abundant (e.g., Figure 1C). Litter samples were shipped to the University of Florida where they were spread in a single layer on a greenhouse bench (25°C) to air dry to constant mass (~1 week). Samples were air‐dried rather than oven‐dried to enhance survival of the resident microbial community.

### Litter bags

Litter bags were made with 18 × 16 (openings horizontally × vertically per square inch) weave DocaScreen fiberglass mesh window screening (Docazoo, Memphis, TN, USA). A 30 cm × 25 cm section of mesh was folded in half lengthwise, forming a 15 cm × 25 cm rectangle. We folded over the edges by 5 mm and stapled along the edge at 3‐cm intervals to form the litter bags. The bags were filled with 10 g of the air‐dried litter and stapled shut. Each bag also contained a numbered aluminum tag (Forestry Suppliers, Jackson, MS, USA) for identification.

**FIGURE 2:**
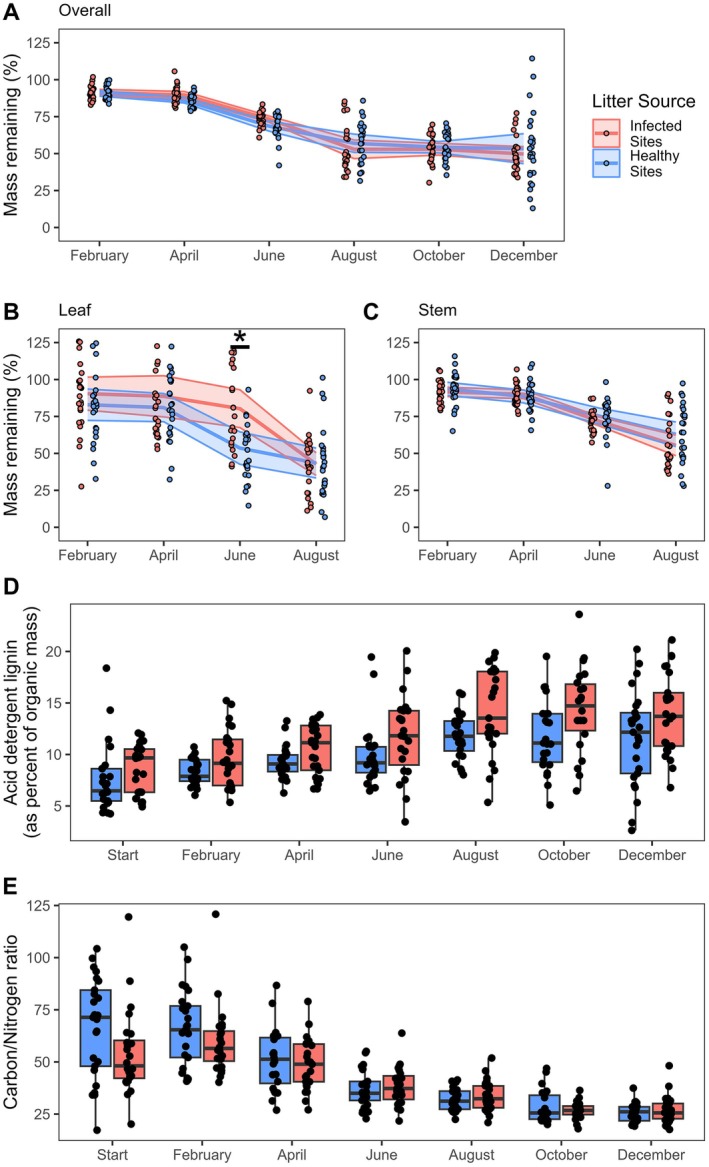
Percent of (A) overall mass, (B) leaf mass, (C) stem mass, remaining at each timepoint. Data points from litter originating from infected sites are offset to the left of each timepoint while points from healthy litter sites are offset to the right. Shaded region represents 95% CI. Stars indicate significant differences between infected and healthy samples, for full statistical results see Table 1 and Appendix S1: Table S8. Litter quality was estimated by the (D) acid detergent lignin content (as percent or organic mass) and (E) the carbon/nitrogen ratio of litter at each timepoint. The acid detergent lignin content was significantly higher in litter from infected sites. Post hoc comparisons were not conducted for litter quality metrics as there was no significant interaction between month and infection (Table 1).

Because litter samples were not oven‐dried, the absolute moisture content of the greenhouse dried litter from each site was estimated by taking five 10‐g samples per site of the air‐dried litter. Each sample was dried at 70°C to constant mass. The resulting dry masses were used to estimate the starting dry mass of the litter bags. In practice, our first litter collection showed that initial dry mass varied among bags because the dry mass of some litter samples in February 2021 was greater than our estimates from December (Figure 2). The initial proportion of stem and leaf composition of each sample, measured as the proportion of dry mass comprised of either stem or leaf material, was determined by separating the stem and leaf tissue of a 2‐g sample, oven drying, and weighing.

### Experiment at common garden site

On December 10, 2019, five blocks (3 m × 6 m) were established at the healthy common garden site with 10 m between each block. Blocks were selected such that senesced, healthy *M. vimineum* was relatively uniform across each block. In each block, seven bags of litter from each site (35 bags from five infected sites, 35 bags from five healthy sites, plus 7 bags from the healthy common garden site) were placed into two plots by infection status of the litter bags. One plot contained litter bags from the infected sites while the other contained litter bags from the healthy sites and the common garden site. Within each plot, litter bags were randomized and placed in a grid with 0.5‐m spacing below the naturally fallen *M. vimineum* litter on the soil surface and held in place by a 10‐cm steel landscape fabric pin (Sta‐Green, Mooresville, NC, USA). Plots containing litter bags from infected and healthy sites were separated by 1.5 m within a block to minimize potential pathogen transmission from infected litter to healthy litter. To track hourly temperature and humidity over the course of the experiment, a HOBO Pro v2 temperature and humidity logger (Cape Cod, MA, USA) was placed in each block above the litter layer (Appendix S1: Figure S1). Rainfall data were obtained from the National Climatic Data Center (Station US1KYTB0005).

### Bag collection and analysis

At two‐month intervals (Appendix S1: Table S2), one replicate bag of each site of origin was collected from each block (11 bags per block per collection date, 55 total bags per collection date). The litter bags were transported to Indiana University where approximately 1 g of litter was removed from each bag and shipped on dry ice to the University of Florida where it was stored at −80°C for nucleic acid extraction. The bags with remaining litter were then shipped to the University of Florida and stored at −20°C for biomass analysis.

Each litter bag was gently brushed to remove dirt and debris and rinsed to remove soil from the interior. A representative 1‐g sub‐sample of tissue (wet mass) was removed from the litter bags and separated into leaf and stem material. The separated leaf and stem material, as well as the remaining biomass material, were then dried at 70°C for 1 week to determine dry mass. Stem and leaf material could not be separately identified for the later October and December 2020 samples due to advanced decomposition. Following biomass measurements, samples were passed through a cyclone sample mill (UDY Corporation, Fort Collins, CO) with a 1‐mm screen. We homogenized the ground tissue and collected 0.5‐g subsamples for the determination of litter carbon/nitrogen ratios at the University of Florida Light Stable Isotope Mass Spectrometry Laboratory. The remainder of the sample was sent to the University of Florida Forage Analysis Lab for the determination of lignin content by acid detergent analysis.

### Statistical analyses

Decomposition at each collection time was measured as the dry mass of the remaining litter relative to the estimated initial dry mass. Tests of decomposition, C:N ratio, lignin content, and microbial alpha diversity metrics between litter from infected and healthy sites at each collection time were conducted using a linear mixed‐effects model with infection status, month (ordinal categorical), and their interaction as fixed effects. Block and the litter source (site from which litter was collected; Appendix S1: Table S1) were used as random effects: *lmer(Response ~ Infection × Month + (1|Block*) *+ (1|Source*)). Normality of the residuals was confirmed through inspection of residual plots, and homogeneity of variance across litter sources at each timepoint was confirmed using Levene's test (Kozak & Piepho, 2018). Data with nonnormal residuals were transformed as necessary. Significant effects of infection status indicated that foliar *Bipolaris* pathogens altered the decomposition of host litter, and significant interactions between infection status and time showed temporal variation in the decomposition of litter from infected and healthy sites. Post hoc comparisons were conducted with the *emmeans* package in R. Effect sizes (calculated as Cohen's D) were calculated with the *cohensD* function in the R package *lsr* (Navarro, 2015). All statistical analyses were conducted in R version 4.0.3 using the packages *car, lme4*, and *lmerTest* (Bates et al., 2015; Fox & Weisberg, 2019; Kuznetsova et al., 2017).

### Molecular methods and sequence processing

Nucleic acids were extracted from subsamples of litter and amplified for Illumina MiSeq 2 × 300 bp sequencing. In brief, litter samples containing both stem and leaf tissue and negative controls were lyophilized, bead beat, and DNA was extracted using CTAB with a glass milk silica matrix used for DNA purification (Lane et al., 2023). The ITS2 region of fungal DNA was amplified using the primers fITS7 and ITS4. Sequences were quality filtered with DADA2 (Callahan et al., 2016) and exported to R for further analyses. The *decontam* package (Davis et al., 2018) was used to process negative controls and remove contaminating sequences. The rarefaction curves of all samples were manually inspected to confirm adequate sequencing depth; all samples containing less than 10,000 high‐quality reads were removed. Full details of DNA extraction and quality filtering may be found in Appendix S1.

For analysis of alpha diversity metrics, samples were rarified to the sequencing depth of the smallest sample. We estimated community richness using the Chao1 index and the Shannon's diversity index using the *estimate_richness* function in the R package *phyloseq*. The Chao1 index is a nonparametric index used to estimate the total number of species in microbial community data (Kim et al., 2017). Pielou's evenness index was calculated as Shannon's diversity index divided by the natural log of the total number of species (as estimated by the Chao1 index) (Pielou, 1966). Alpha diversity metrics were compared using a linear mixed‐effects model with infection status, month, and their interaction as fixed effects with block and litter source as random effects.

For analyses of community composition, amplicon sequence variants (ASVs) were scaled to relative abundances within samples without rarefaction. Principal coordinates analysis (PCoA) ordination plots were calculated using the *pcoa* function in *ape* using both Bray–Curtis and Jaccard distances (Paradis & Schliep, 2019). We tested the impacts of litter infection at each timepoint and change over time on microbial community composition using Bray–Curtis and Jaccard distances using permutational multivariate analysis of variance (PERMANOVA) with the *adonis* function using 10,000 permutations. We tested the differential abundance of individual ASVs at each timepoint using ANCOMBC2 (Lin & Peddada, 2024) with a Holm multiple comparison correction as calculated by ANCOMBC2. All analyses using ANCOMBC2 were conducted without scaling to relative abundances (using the count data) as ANCOMBC2 conducts its own data transformations. We also implemented a minimum natural log fold change threshold (by infection status) of two, as calculated by ANCOMBC2. Each differentially abundant ASV was functionally categorized as “Plant‐associated,” “Saprophytic,” “Both,” or “Other” through manual inspection of the FUNGuild database and literature searches (Nguyen et al., 2016). ASVs which were not identified to at least the family level were functionally categorized as “Unknown.” Differential abundance analyses using ANCOMBC2 were conducted between infected and healthy litter and within timepoints. We did not use ANCOMBC2 to quantify changes over time due to potential limitations in the detection of rare organisms (immigration vs. growth of an existing organism), the inability of ITS sequencing to differentiate between dormant and active microbes, and due to the known ability of DNA from dead organisms to persist within the environment which hampers the ability to determine when a fungal taxa has gone extinct from the community (Carini et al., 2016).

## RESULTS

### Decomposition of total litter from infected and healthy sites

Over the course of 1 year (December 2019 to December 2020), the litter bags lost an average of 48.5% ± 2.7% (mean ± SE) of their total mass (Figure 2A; Appendix S1: Table S3). Most decomposition took place between April and August collection dates, accounting for nearly three‐quarters of the total mass lost. While time was a significant predictor of decomposition, we did not observe a significant difference in the proportion of overall mass remaining between litter from healthy and infected sites nor an interaction between sampling timepoint and infection status for the proportion of overall mass remaining (Table 1).

**TABLE 1:** ANOVA examining the impact of infection and month on proportion of litter mass remaining, acid detergent lignin content, and carbon:nitrogen ratio.

Dependent variable	Tissue	Transformation	Factor	NumDF	DenDF	*F* value	*p* value
Mass remaining	Overall	None	Infection	1	8.20	0.00	0.9971
			Month	5	270.64	142.5276	<0.001
			Infection:Month	5	270.65	1.4484	0.2072
	Leaf	Square root	Infection	1	7.97	2.0716	0.18817
			Month	3	175.13	42.6468	<0.001
			Infection:Month	3	175.14	3.1206	0.02741
	Stem	Squared	Infection	1	7.941	1.1348	0.3181
			Month	3	175.05	94.9048	<0.001
			Infection:Month	3	175.06	1.1493	0.3307
Lignin	Overall	Cubic root	Infection	1	8.00	2.5246	0.1508
			Month	6	313.15	24.7818	<0.001
			Infection:Month	6	313.17	0.2944	0.9394
C:N ratio	Overall	Log	Infection	1	7.98	0.0519	0.8256
			Month	6	313.057	130.2599	<0.001
			Infection:Month	6	313.086	2.0301	0.0614

*Note*: Data transformations for each analysis are indicated in the table. Degrees of freedom were calculated with Satterthwaite's method.

### Leaf and stem decomposition from infected versus healthy sites

Infected and healthy litter dried mass was initially comprised of 19.0% ± 1.79% and 29.1% ± 2.24% (mean ± SE) leaf material, respectively. The sampling timepoint and the interaction between infection status and sampling timepoint significantly influenced the decomposition of leaf material. Post hoc analysis revealed 53.6% ± 5.30% of leaf material from healthy sites remained undecomposed in June, which was significantly less than leaf material from infected sites (80.3% ± 6.16%) (Tukey post hoc *p* = 0.0002; Cohen's *D* = 1.006). There were no significant differences in the decomposition of leaf material by infection status at other collection dates (Figure 2B). The sampling timepoint, but not infection status or their interaction, influenced the decomposition of stems (Figure 2C, Table 1). By August, litter samples had lost 56.9% ± 3.01% of their total leaf mass and 40.9% ± 2.6% of their stem mass in total with no significant differences by infection status.

### Litter chemical composition

Lignin composition (as a proportion of organic mass) was impacted by the sampling timepoint, but not by infection status or the interaction of infection status and time. The lignin content of decomposing litter steadily increased at each timepoint from the initial collection until August (Figure 2D; Appendix S1: Table S4). The initial C:N ratio of healthy *M. vimineum* litter was 67.6 (Figure 2E; Appendix S1: Table S4), consistent with previous reports of naturally senesced *M. vimineum* (DeMeester & Richter, 2010). The C:N ratio of litter was significantly impacted by the sampling timepoint, continuously decreasing over the course of the experiment. We observed no significant differences in the C:N ratio of litter from infected and healthy sites.

### Microbial community analysis

**FIGURE 3:**
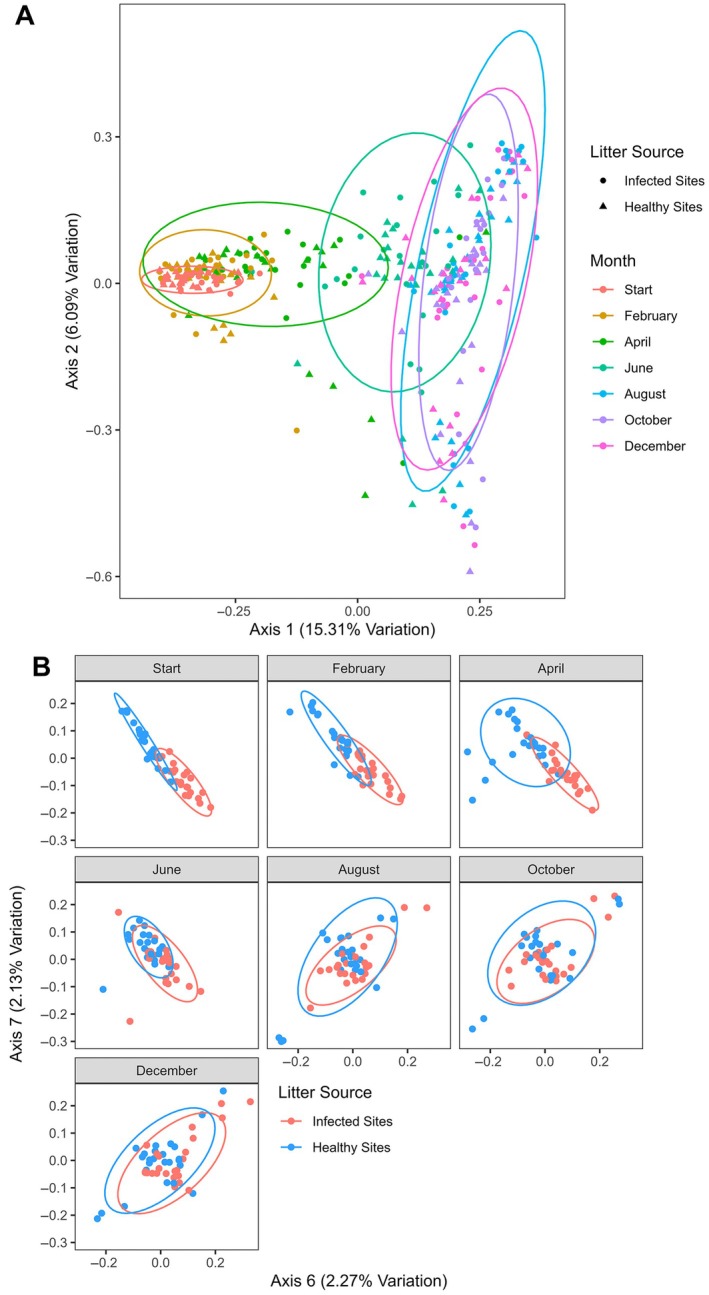
(A) Principal coordinates analysis (PCoA) of microbial populations using Bray–Curtis distances. Ellipses are drawn for each timepoint regardless of infection status. (B) PCoA of microbial populations using Bray–Curtis distances and axes 6 and 7, which best represent the separation of the microbial communities by litter source. Full permutational multivariate analysis of variance results of each comparison can be found in Appendix S1: Table S6.

**FIGURE 4:**
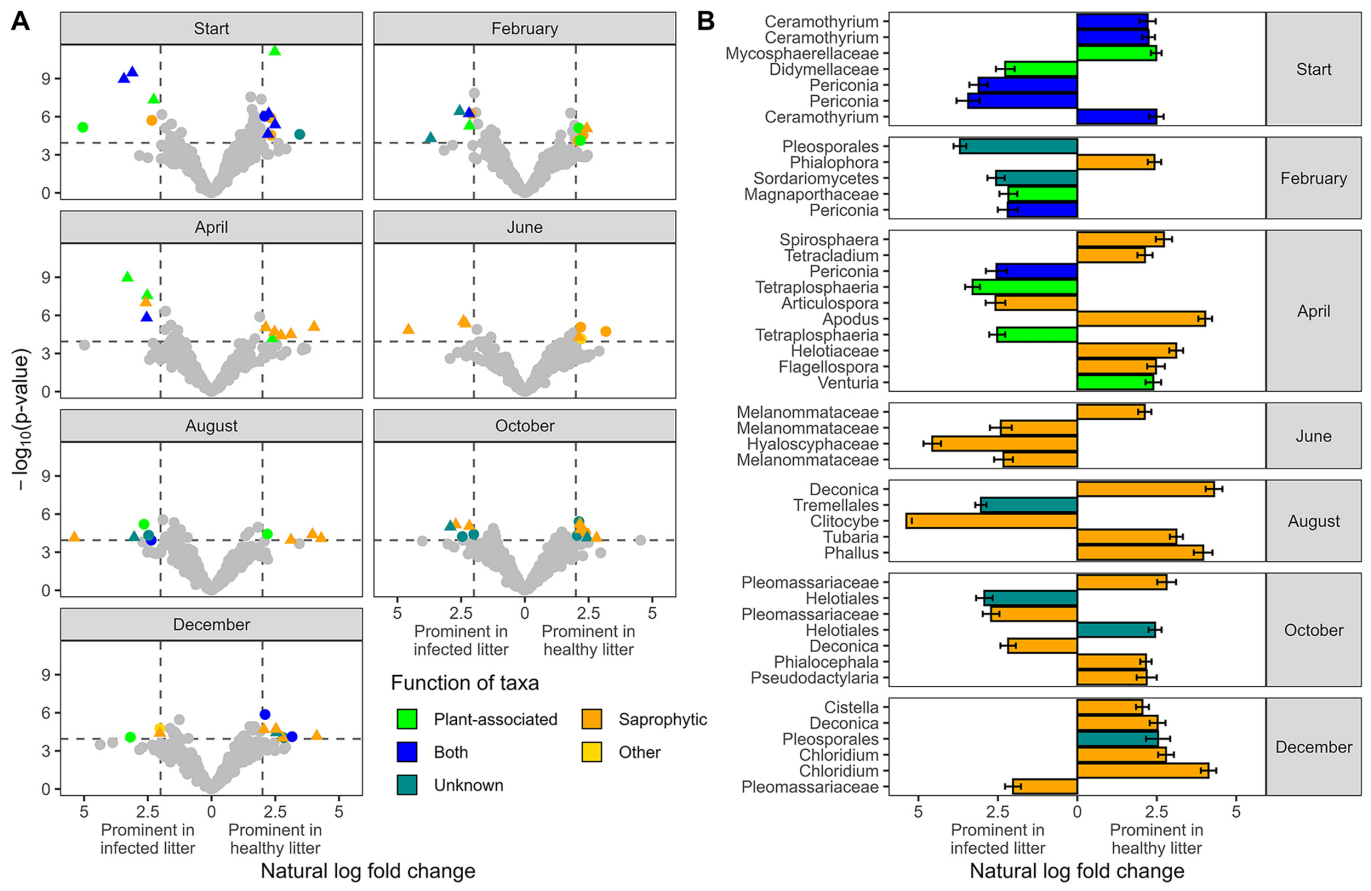
(A) Differentially enriched amplicon sequence variants (ASVs) in infected litter (left in the plot) and healthy litter (right in plot). Dot color indicates the estimated function of each ASV, ASVs that could not be identified to at least the family level were labeled as unknown function. Triangular dots indicate ASVs which comprised at least 0.05% of the reads for each timepoint. Gray dots indicate ASVs which were not significant following a Holm correction (dashed horizontal lines) or did not undergo a natural log fold change of at least two (dashed vertical lines) by infection status. (B) Natural log fold change by timepoint of ASVs which contained at least 0.05% of the reads. Bars represent standard error. All statistics for both panels were calculated using ANCOMBC2 and can be found in Appendix S1: Table S8.

**FIGURE 5:**
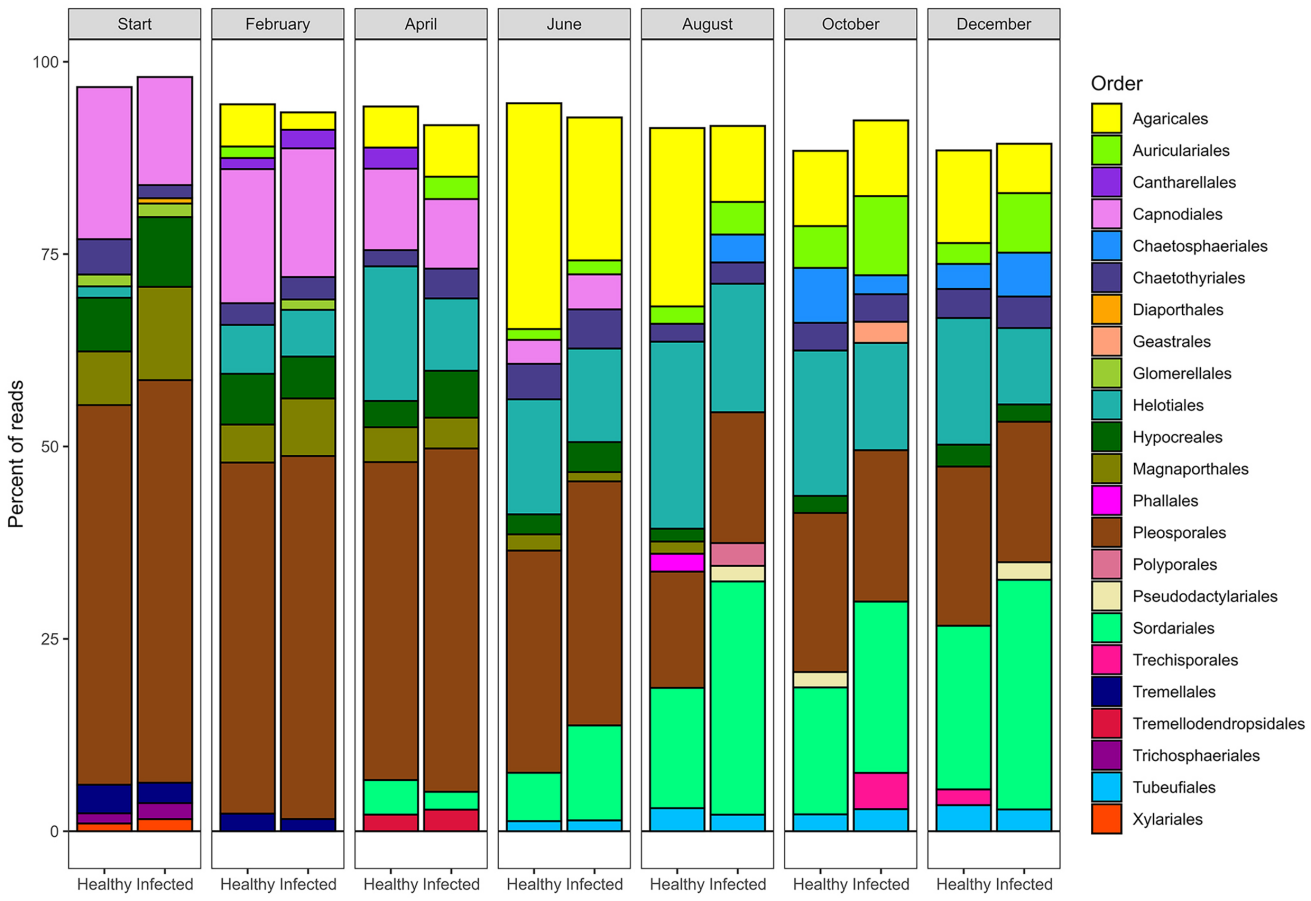
Top 10 most abundant fungal orders for each infection status and timepoint. Bars within each panel are retained in the same order as the legend.

Healthy litter had a significantly higher Shannon's diversity and Pielou's evenness of fungal communities at the December timepoint (Tukey post hoc, *p* = 0.0161 and *p* = 0.0048, respectively; Appendix S1: Table S5). All three indices were lowest at the August collection, except for Pielou's evenness, which was lowest at the end of the experiment in December 2020 (Appendix S1: Table S4). Microbial community analysis revealed a gradual shift in community composition during the first 6 months of the experiment. Infection status significantly impacted community composition from the start of the experiment through August with the exception of the June collection (Appendix S1: Table S6). From the August collection onward, there were no significant changes in the community composition of infected or healthy litter between timepoints (Figure 3A; Appendix S1: Table S7). Differences in the microbial communities by litter infection status could be most clearly visualized using PCoA axes 6 and 7 and showed convergence over time (Figure 3B). Analysis of ASVs at each timepoint revealed a total of 72 ASVs which were differentially abundant by infection status and had a minimum natural log fold change of two (range 7–13 for each sampling date) (Figure 4A; Appendix S1: Table S8). When we added the criterion that the ASV must contain at least 0.05% of the reads for that timepoint, there were 44 differentially abundant ASVs (Figure 4B). In our comparison of top 10 orders by infection status/timepoint, we observed a change in the most prominent orders over time (Figure 5). The variation in the relative abundances of prominent orders became more pronounced over the course of the experiment. By August, the Auriculariales and Sordariales comprised a larger proportion of the reads from infected litter while Agaricales and Helotiales comprised a larger proportion of the reads from healthy litter (Figure 5).

### Decomposition of litter from experimental site

Healthy litter originally obtained from the common garden site was significantly more decomposed than the translocated healthy litter (Appendix S1: Figure S2, Table S9). By December, translocated healthy litter had 35.58% more biomass than healthy litter obtained from the common garden site (Cohen's *D* = 0.69; Appendix S1: Table S4). The differences in the decomposition of healthy litter obtained from the common garden site and translocated healthy litter could not be attributed to either the stem or leaf material (Appendix S1: Table S9). The lignin content of healthy litter was influenced by sampling timepoint, but not influenced by the origin of litter (translocated vs. experimental common garden site) or the interaction of litter origin and time. The C:N ratio was 44.08% lower in the litter from the experimental site at the start of the experiment (Cohen's *D* = 1.24; Appendix S1: Table S4) and was significantly impacted by the sampling timepoint as well as the interaction of litter origin and the sampling timepoint (Appendix S1: Table S9). However, post hoc analyses were not able to identify significant differences in the C:N ratio of litter from the experimental site and translocated healthy litter at any sampling timepoint. The microbial communities of translocated healthy litter and litter obtained from the common garden site were significantly different in composition early in the experiment but had converged by the February sampling based on Jaccard distances and by the April sampling based on Bray–Curtis distances (Appendix S1: Table S6). The significant Bray–Curtis distances, but not Jaccard distances, at the February sampling suggest similarity in membership, but not the differential abundances of prominent members of the community. There were no significant differences in diversity metrics between common garden and translocated healthy litter at any timepoint (Appendix S1: Table S5). We did not conduct direct comparisons between translocated infected litter and healthy litter originating from the experimental common garden site as the effects of infection and litter translocation would be confounded.

## DISCUSSION

We hypothesized that pathogens affect litter decomposition by altering the chemistry of host litter. Our results revealed that the establishment of a foliar pathogen on an invasive annual grass shifted the timing of decomposition of infected leaf tissue from spring (April to June) to summer (June to August), but we did not detect differential host lignin nor C:N content. Rather, alterations to decomposition following pathogen colonization may arise from disease‐associated shifts in the microbial community.

Decomposition of leaf litter from infected sites coincided with increased average daily high temperatures (June–August mean daily high 26.0°C). When not limited by moisture availability, early‐stage decomposition of graminicolous litter is driven primarily by temperature, the optimal temperature being approximately 23°C (Coûteaux et al., 2002; Dalias et al., 2001). Prior to the occurrence of these optimal temperatures in mid‐April (Appendix S1: Figure S1), we observed a low rate of leaf decomposition across all samples and no significant differences between infected and healthy samples. Litter quality, which is defined as how rapidly different litter is decomposed under identical conditions (faster decomposition = higher quality), is largely determined by carbon and nitrogen composition (a lower C:N ratio decomposes more rapidly) as well as overall carbon recalcitrancy (recalcitrant carbon is slow to metabolize) (Adair et al., 2008; Parton et al., 1994; Taylor et al., 1989). Fierer et al. (2005) found that higher quality litter is more rapidly decomposed at a wider range of temperatures than lower quality litter and higher daily temperatures are more conducive for the decomposition of substrates with an elevated lignin content. We expected that disease could cause localized lignification resulting from a host defense cascade, potentially reducing the quality of the litter and slowing decomposition at earlier, cooler timepoints (Ayres, 1993; Bhuiyan et al., 2009; Hammerschmidt & Schultz, 1996; Rahman et al., 2013). However, we measured lignin from the whole sample material of leaves and stems, whereas *Bipolaris* pathogens of *M. vimineum* do not cause disease on stem tissue (Lane et al., 2020; Manamgoda et al., 2013; Minnis et al., 2012). We suspect alterations to lignin content may have occurred in foliar tissue, where we observed disease‐associated effects on decomposition, but we were unable to detect lignification across the whole sample as it was dominated by stem biomass. The C:N ratio of litter from infected and healthy sites was not significantly different, indicating the C:N ratio did not play a significant role in differential decomposition of litter by infection status.

We found significant differences in fungal communities between litter types at each collection time up to August, except for the June collection (Appendix S1: Table S6). In June, we observed especially high variation in microbial communities among samples (elevated median distance to centroid, Appendix S1: Table S10) and a shift in microbial community composition (Figure 3A). The decomposition of litter mass does not typically occur at a constant rate; rather, initial decomposition occurs rapidly as exponential decay, and the rate of mass loss slows through the early phase of decomposition (Peng et al., 2014; Swift et al., 1979). Early in the litter decomposition process, microbial communities feed on readily available nutrient sources, resulting in an initial rapid loss in dry mass (Berg & McClaugherty, 2003). Following the depletion of readily available carbohydrates, microbial communities must metabolize increasingly complex substrates, subsequently slowing the rate of decomposition (Berg & McClaugherty, 2003; Peng et al., 2014). The utilization of increasingly complex substrates is associated with shifts in the microbial assemblage (Veen et al., 2019) such as those observed in this study (Figure 3A). In our experiment, there were no significant differences in the leaf mass of litter from infected and healthy sites by August, and there was little loss of litter mass after August when temperatures were still optimal for microbial growth and decomposition. By October, the overall degradation of samples had progressed to the point that we were no longer able to differentiate stem and leaf material within the litter bags. Moreover, lignin composition, C:N ratios, and microbial communities of our samples began to stabilize starting with the August samples; all these observations suggest the depletion of labile carbohydrates and the concurrent transition of microbial communities to the metabolism of primarily complex substrates.

While temporal dynamics of litter decomposition play the largest role in microbial community composition (Gołębiewski et al., 2019; Tláskal et al., 2016; Voříšková & Baldrian, 2013), our results suggest that establishment of a foliar pathogen prior to senescence can have a persistent role in the trajectory of microbial decomposer communities. The history of fungal community assembly can be critical in the decomposition of litter material (Cline & Zak, 2015). Early colonizers may modify their habitat and alter later emerging assemblages (Lane et al., 2023; Vannette & Fukami, 2014). In our study system, *B. gigantea* produces several herbicidal ophiobolins with potential antimicrobial activity (Evidente et al., 2006; Li et al., 1995). The induction of chlorophyll synthesis within *B. gigantea* lesions, sometimes referred to as the “green island effect”, indicates the potential production of secondary metabolites which may alter microbial communities (Lane et al., 2020). In addition, pathogen colonization may exclude the natural microbial communities that are involved in rapid decomposition of *M. vimineum* litter through resource competition, priority effects, or even indirectly, such as through holistic shifts in the plant‐associated community resulting from growth‐defense trade‐offs (Adame‐Álvarez et al., 2014; Lane, Kuhs, et al., 2025; Noman et al., 2021). Differences in fungal communities in the spring appeared to have downstream impacts on the most prominent fungal orders by the end of the experiment. Differentially enriched ASVs in the translocated healthy litter were primarily identified as saprophytic taxa by April, while the differentially enriched taxa in infected litter continued to be primarily plant‐associated pathogens and endophytes. These differences in the microbial community may in part explain the differential decomposition of leaf tissue and could have significant potential impacts on ecological functions including alterations to the metabolism of carbon substrates and the profile of greenhouse gasses released (Popp et al., 2017), the availability of nitrogen compounds within the soil (Wang et al., 2021), and germination of seeds.

Litter is degraded most rapidly at the site of origin in a phenomenon often dubbed, ‘home‐field advantage’ (Ayres et al., 2009), thus necessitating the use of common garden experiments. Here we observed that healthy litter from the experimental common garden site decomposed more rapidly than translocated healthy litter, confirming that using a common garden site to which both infected and healthy litter were translocated was essential for the experiment. Notably, differences between microbial communities of healthy locally sourced litter and translocated healthy litter persisted only through the April collection, while differences between translocated infected and translocated healthy litter persisted through August (Appendix S1: Table S6). Home‐field advantage has been linked to competition between allopatric soil‐inhabiting decomposers, which are colonizing the translocated litter, and native litter‐microbial communities. Non‐native plant species may leave behind their coevolved microbial communities when they establish in a new region (Mitchell & Power, 2003). However, due to the complex dynamics which govern the assembly of native microbes on non‐native plant species, it was unclear whether invasive *M. vimineum* decomposition would reflect home‐field advantage. Our results suggest *M. vimineum* (with implications for other non‐native plant species) develops an assemblage of endo‐ and epiphytic fungi (Lane et al., 2023) and forms native litter‐microbial communities which may compete with soil‐inhabiting decomposers following translocation, consistent with the hypothesized mechanisms underlying home‐field advantage in native plant systems. While we cannot exclude the possibility that soil‐microbial communities of infected sites would be better suited for the decomposition of infected litter (and vice‐versa), *M. vimineum* is known to spread via floodwaters that translocate senesced plant material with their seeds, emphasizing the ecological relevance of the decomposition dynamics of translocated litter of invasive plant species (Barden, 1987). Natural translocation could also bring *B. gigantea* infected *M. vimineum* to non‐diseased *M. vimineum* populations, highlighting the importance of the decomposition dynamics of infected litter in healthy plant populations.

Pathogen impacts on litter dynamics could alter epidemic development, because plant litter contributes to the survival and dispersal of fungal inoculum between seasons (Knudsen et al., 1995; Neate, 1987; Suffert et al., 2011). Optimal temperatures for germination and growth of *Bipolaris* pathogens occur in the summer months (Almaguer et al., 2013). Positive feedback on disease could arise from slowed decomposition, in which pathogen‐mediated persistence of infected litter promotes the survival and dispersal of primary inoculum the following season. Alternatively, relative increases in the composition of recalcitrant carbon (such as those we hypothesize occurred in the leaf material) may lead to antibiosis‐mediated competition for recalcitrant carbon, potentially reducing the survival of pathogenic inoculum within the litter (Lane, Tran, et al., 2025).

These results have important implications for our growing understanding of relationships between plant disease dynamics and nutrient cycling (Paseka et al., 2020). Pathogen colonization may increase host mortality and thereby alter nutrient dynamics by accelerating the rate at which nutrients are returned to the environmental pool (Borer et al., 2022). Our work demonstrates marked differences in the seasonal phenology of litter decomposition, depending on the infection status of the litter. The effect of the pathogen on decomposition is transient, but the timing of the delay in decomposition comes at a critical juncture for both plant and pathogen. As the growing season progresses, *M. vimineum* populations become dense and allocate resources to rapid vegetative growth, which may indicate more intense resource competition (De Wit, 1960) and amplify potential impacts of delayed decomposition on nutrient availability. Different temporal patterns in decomposing litter are expected to have different timing of release of nutrient pulses and magnitudes of litter impacts on germination. Indeed, many plants have narrow temporal windows for germination and early development. There is increasing attention being given to seasonality in analyses of ecological systems (White & Hastings, 2020), and our results suggest that infection influences the phenology of decomposition. Together, these findings suggest foliar fungal pathogens may contribute opposing forces to nutrient cycling: speeding senescence but slowing decomposition.

Our results demonstrate for the first time that fungal pathogen colonization alters litter decomposition phenology with potential wide‐ranging ecosystem effects including nutrient cycling and the development of subsequent epidemics. Moreover, the impact of pathogens on litter decomposition could interact with plant competition to alter plant community composition, with possibly surprising feedback effects on litter decomposition (Kortessis et al., 2022). While this study was conducted in a single system, the interplay of disease dynamics and litter decomposition processes is likely to be important in many plant‐pathogen systems. Rising global temperatures and increasing atmospheric CO_2_ are anticipated to expand pathogen range and disease severity in both natural and agronomic systems (Raza & Bebber, 2022). The expansion of pathogen ranges to include new potential hosts may subsequently have wide‐ranging impacts on ecosystem processes—including decomposition dynamics, nutrient cycling, the flow of energy through food webs, and carbon sequestration—further exacerbating the impact of rising global temperatures on ecosystem health.

## CONFLICT OF INTEREST STATEMENT

The authors declare no conflicts of interest.

## Supporting information


Appendix S1.

## Data Availability

All sequencing data are available in the National Center for Biotechnology Information (NCBI) Sequence Read Archive under accession number PRJNA978091 at https://www.ncbi.nlm.nih.gov/bioproject/PRJNA978091. All other data (Lane, 2023) are available in the Environmental Data Initiative (EDI) at https://doi.org/10.6073/pasta/d148c1ea2ae5bc8a9ec4403c34027d35. Code (Lane, 2026) is available in Zenodo at https://doi.org/10.5281/zenodo.18258613.
