# Supplementary material for: Foliar pathogen epidemic slows decomposition of invasive plant litter
Source: Ecology. 2026 Apr 9;107(4):e70374. doi: 10.1002/ecy.70374 (PMC13063362; doi:10.1002/ecy.70374)
Supplement: Supplementary file 1 — Appendix S1. [file ECY-107-e70374-s001.pdf]

Foliar pathogen epidemic slows decomposition of invasive plant litter

Brett R. Lane, Chris Wojan, Carlie Meehan, Amy E. Kendig, Robert D. Holt, Philip F. Harmon, Keith Clay, S. Luke Flory, Erica M. Goss

Ecology

Appendix S1

Molecular methods and sequence processing

Subsamples of litter collected for nucleic acid extraction were lyophilized in a FreeZone 2.5 Plus (Labconco Corporation, Kansas City, MO). A 0.25 g subsample of each, containing both leaf and stem tissue, was placed in a 1.5 mL screw open tube with 3 sterile 4.5mm metal beads (Daisy Outdoor Products, Rogers, AR) and beaten for 60 seconds in a Fastprep-24 bead beater (MP Biomedicals, Solon, OH), or until each sample was powdered. Following bead beating, 750 µL of CTAB extraction solution (Teknova, Hollister, CA) was added to each tube. The tubes were then vortexed and placed at -20°C overnight. We extracted DNA as described by (Brazee and Lindner 2013). Briefly, tubes were placed in a water bath at 65°C for 2 hours then pelleted by centrifugation at 10,000 rfc for 1 minute. We transferred 100 µL of supernatant into strip tubes for DNA extraction. We precipitated the DNA with 150 uL of ice-cold isopropanol and pelleted the precipitate by centrifugation at 10000 rfc for 20 minutes. We washed the pellet with 175 µL of 70% molecular grade EtOH. The DNA was bound to 2.5 µL of glass milk in a solution of 45 µL of molecular grade water, and 135 µL of NaI solution. Bound DNA was washed with 175 µL of wash buffer and eluted in 50 µL of molecular grade water. The glass milk, NaI solution, and wash buffer were obtained from the GeneClean II kit (MP Biomedicals, Irvine, CA). An aliquot of the above extracted DNA was diluted 1:20 for PCR amplification with nucleotide free water. We amplified the ITS2 segment of the internal transcribed spacer (ITS)

using primers fITS7 and ITS4 (White et al. 1990, Ihrmark et al. 2012) modified with Illumina Nextera v2 adapters (Illumina Inc., San Diego, CA). Each reaction contained 7.5 µL of 2x GoTaq Green Master Mix (Promega, Madison, WI), 0.15 µL BSA (New England Biolabs, Ipswich, MA), primers to a final concentration of 0.2µM, 2 µL of the above diluted DNA, and molecular grade water to 15µL. The PCR conditions, obtained from Lane et al. (2023), were 94°C for 3 minutes, followed by 37 cycles of 94°C for 30 seconds, 60°C for 30 seconds, stepping down 0.5°C each cycle until reaching 55°C, and an extension at 72°C for 1 minute. The final extension was conducted at 72°C for 7 minutes. Amplification was confirmed by agarose gel visualization. A second step of PCR amplification was conducted to dual index the above product using Illumina Nextera v2 indices and adapters (Illumina Inc., San Diego, CA). The reactions were identical to the preceding PCR, except annealing at 55°C for eight cycles. Indexed samples were quantified by comparison of bands on agarose gels to Low DNA Mass Ladder (Fisher Scientific). In addition to our samples, we also amplified negative controls from each step of DNA extraction and amplification. Each negative control was carried through subsequent steps. The indexed samples were grouped by concentration in sets of three for removal of primers, dNTPs, salts, and other post-PCR contaminants using Zymo Select-A-Size spin columns (Zymo Research, Irvine, CA). The purified, indexed DNA was quantified using an Invitrogen Qubit 4 fluorometer and combined into two amplicon libraries in equimolar concentrations. Negative samples were included in the libraries using the median volume of all other samples. The amplicon libraries were shipped overnight to the University of California – Riverside, Institute for Integrative Genome Biology, for sequencing using 2 x 300 bp sequencing on an Illumina MiSeq.

Demultiplexed ITS2 amplicon reads were imported into the open-source pipeline, Quantitative Insights Into Microbial Ecology 2 (QIIME2) (Bolyen et al. 2019). Briefly, we trimmed adapters and primers before denoising and filtering the sequences using DADA2 (Callahan et al. 2016). The leading 25 bp were trimmed due to poor quality and reads were truncated at 350 bp for a total amplicon length of 325 bp. Taxonomic assignment was performed using a naïve-Bayes classifier trained on the UNITE database version 8.3 including all eukaryotic sequences (Nilsson et al. 2019). Data was then transferred to R version 4.0.3 for subsequent analyses using the packages *vegan* and *phyloseq* (McMurdie and Holmes 2013, Oksanen et al. 2022). All amplicon sequence variants (ASVs) identified as non-fungal were discarded from further analyses. The *decontam* package (Davis et al. 2018) was used to process negative controls and remove contaminating sequences. All samples containing less than 10,000 high-quality reads were removed. Adequate sequencing depth for the remaining samples was confirmed via manual inspection for saturation of rarefaction curves.

Table S1. Sites of litter collection used in this study

	Latitude	Longitude
Experimental site	38.9781	-85.3632
Infected 1	38.9873	-85.3794
Infected 2	38.9715	-85.3639
Infected 3	38.9656	-85.3645
Infected 4	38.9625	-85.3651
Infected 5	38.9752	-85.4675
Healthy 1	38.9871	-85.3773
Healthy 2	38.9893	-85.3617
Healthy 3	38.9773	-85.3631
Healthy 4	38.9434	-85.4625
Healthy 5	38.9640	-85.4632

Table S2. Litter bag collection dates and summary of climate data over the course of the experiment. Temperature and humidity data was collected by data loggers place within plots. Precipitation data were obtained from the National Climatic Data Center (Station US1KYTB005).

Month	Litter Bag Collection Date	Average High (°C)	Average Low (°C)	Average Relative Humidity	Total Precipitation (cm)
December 2019†	December 10	8.9	-1.7	88	7.4
January 2020	-	6.7	-1.2	88	9.2
February 2020	February 24	6.7	-3	87	10.2
March 2020	-	14.7	3.3	83	13.7
April 2020	April 24	17.9	4.9	77	10.7
May 2020	-	20.3	11.1	86	13.8
June 2020	June 25	25.8	17	87	13.1
July 2020	-	27.6	19.9	94	18.8
August 2020	August 25	24.6	18.1	96	13.7
September 2020	-	23	13.6	91	5.5
October 2020	October 28	17.6	7.5	89	14.1
November 2020	-	17	1.8	82	7.6
December 2020†	December 15	8.2	-2.4	89	1.1

†Weather data was only collected while the experiment was ongoing

Table S3. Percent mass remaining overall and of leaf or stem tissue by collection date.

		Percent Litter Remaining		
		Infected (translocated)	Healthy (translocated)	Experimental site
Overall	February	91.4 (0.99)†	90.7 (0.88)	89.0 (2.93)
	April	89.7 (1.13)	86.1 (0.88)	74.8 (1.42)
	June	73.3 (1.03)	68.6 (1.65)	51.3 (6.68)
	August	52.9 (3.04)	57.0 (2.98)	41.3 (4.77)
	October	52.9 (1.92)	54.3 (1.85)	41.4 (6.13)
	December	49.7 (2.42)	53.3 (4.91)	36.7 (9.06)
Leaf	February	90.4 (5.41)	82.9 (5.14)	78.8 (10.96)
	April	88.6 (6.75)	81.0 (4.61)	56.2 (4.63)
	June	80.3 (6.16)	53.6 (5.30)	18.3 (6.49)
	August	42.7 (3.70)	43.5 (4.88)	13.8 (3.08)
Stem	February	91.6 (1.51)	93.7 (2.15)	95.3 (5.26)
	April	90.0 (1.42)	88.5 (2.08)	86.3 (3.97)
	June	71.7 (1.54)	75.2 (2.62)	71.8 (8.12)
	August	55.4 (3.36)	62.9 (4.02)	58.3 (6.36)

† mean (se)

Table S4. Acid detergent lignin content, Carbon:Nitrogen Ratio, and three alpha diversity metrics (Chao1, Shannon's Diversity, and Pielou's Evenness) at each timepoint for infected and healthy translocated litter as well as litter collected from the experimental site.

		Lignin	CN Ratio	Chao	Shannon	Pielou
Infected	Start	8.63 (0.49)†	54.11 (4.17)	219.67 (10.97)	3.75 (0.073)	0.70 (0.012)
	February	9.63 (0.58)	59.85 (3.36)	236.26 (11.40)	4.05 (0.078)	0.75 (0.011)
	April	10.49 (0.48)	49.11 (2.55)	226.78 (11.63)	4.15 (0.074)	0.77 (0.013)
	June	11.75 (0.82)	38.32 (1.92)	230.54 (13.98)	3.88 (0.125)	0.72 (0.018)
	August	14.23 (0.82)	33.39 (1.43)	180.63 (11.70)	3.17 (0.100)	0.62 (0.016)
	October	14.37 (0.84)	26.83 (0.85)	252.08 (12.94)	3.48 (0.131)	0.64 (0.020)
	December	13.86 (0.78)	26.71 (1.33)	206.80 (9.52)	3.23 (0.130)	0.61 (0.021)
Healthy	Start	7.54 (0.66)	66.75 (4.78)	267.83 (12.80)	3.90 (0.073)	0.71 (0.012)
	February	8.27 (0.26)	66.04 (3.64)	233.67 (12.34)	3.92 (0.072)	0.73 (0.011)
	April	9.27 (0.32)	51.41 (2.98)	221.90 (10.55)	4.06 (0.071)	0.76 (0.011)
	June	9.88 (0.60)	36.77 (1.78)	206.03 (14.60)	3.69 (0.140)	0.70 (0.020)
	August	11.78 (0.44)	31.86 (1.06)	201.86 (16.35)	3.45 (0.097)	0.66 (0.013)
	October	11.77 (0.70)	28.29 (1.56)	274.68 (17.67)	3.76 (0.130)	0.68 (0.019)
	December	11.56 (0.92)	25.75 (0.91)	223.20 (13.71)	3.62 (0.128)	0.68 (0.022)
Common Garden	Start	7.13 (0.13)	37.33 (1.67)	258.18 (21.66)	3.84 (0.124)	0.70 (0.014)
	February	10.89 (0.49)	38.94 (2.60)	188.44 (34.55)	3.63 (0.186)	0.71 (0.010)
	April	12.98 (0.56)	30.62 (1.31)	217.28 (20.98)	4.20 (0.135)	0.79 (0.014)
	June	12.67 (1.60)	26.85 (1.91)	182.56 (11.92)	3.69 (0.370)	0.71 (0.063)
	August	13.27 (0.50)	25.87 (2.26)	168.70 (18.93)	3.50 (0.221)	0.69 (0.032)
	October	15.81 (2.62)	21.90 (0.68)	272.28 (12.04)	3.79 (0.205)	0.68 (0.034)
	December	11.01 (2.06)	20.78 (1.03)	211.23 (33.70)	3.77 (0.257)	0.71 (0.040)

† mean (se)

Table S5. Results of ANOVA indicating impact of liter infection status and month of sampling on alpha diversity metrics. Data transformations for each analysis are indicated in the table. Degrees of freedom were calculated with Satterthwaite's method.

			Transformation	NumDF	DenDF	F-value	p-value
Infected vs translocated healthy	Chao1	Infection†	Square root	1	7.95	0.42	0.535
		Month		6	113.45	7.01	<0.001
		Infection:Month		6	253.13	1.53	0.169
	Shannon's Diversity	Infection	Cubed	1	7.68	1.80	0.218
		Month		6	240.02	14.82	<0.001
		Infection:Month		6	300.99	2.47	0.0240
	Pielou's Evenness	Infection	Cubed	1	314.00	1.50	0.222
		Month		6	314.00	18.34	<0.001
		Infection:Month		6	314.00	2.51	0.0220
Experimental site vs translocated healthy	Chao1	Site†	None	1	4.01	0.58	0.488
		Month		6	138.88	4.08	<0.001
		Site:Month		6	161.71	0.25	0.958
	Shannon's Diversity	Site	Squared	1	4.02	0.0024	0.963
		Month		6	181.23	2.63	0.0183
		Site:Month		6	181.23	0.36	0.904
	Pielou's Evenness	Site	Squared	1	185	0.46	0.498
		Month		6	185	3.31	0.00401
		Site:Month		6	185	0.32	0.928

†Infection is for the comparison of infected and health litter (upper half) while Site [indicating local (from experimental site) vs translocated litter source] is for the comparison of litter from the experimental site against healthy translocated litter (lower half).

Table S6. Results of month-by-month PERMANOVAs comparing the microbial communities of healthy vs infected litter (top) and healthy litter vs litter originating from the experimental site (bottom). Comparisons were conducted using the function *adonis2* using 10,000 permutations.

Comparison	Distance Metric	Month	DF	Residual DF	R^2	F-value	p-value	q-value†
Healthy vs Infected	Bray-Curtis	Start	1	43	0.119	5.80	<0.001	<0.001
		February	1	44	0.065	3.048	<0.001	<0.001
		April	1	46	0.064	3.17	<0.001	<0.001
		June	1	43	0.035	1.56	0.0259	0.181
		August	1	46	0.042	2.0028	0.00390	0.0273
		October	1	45	0.029	1.34	0.0649	0.454
		December	1	47	0.026	1.25	0.119	0.836
	Jaccard	Start	1	43	0.088	4.15	<0.001	<0.001
		February	1	44	0.049	2.29	<0.001	<0.001
		April	1	46	0.049	2.35	<0.001	<0.001
		June	1	43	0.03	1.32	0.0392	0.274
		August	1	46	0.036	1.70	0.00250	0.0175
		October	1	45	0.026	1.19	0.0762	0.533
		December	1	47	0.024	1.15	0.126	0.881
Common Garden vs Healthy	Bray-Curtis	Start	1	26	0.091	2.61	0.00300	0.0210
		February	1	25	0.07	1.88	0.00500	0.0350
		April	1	27	0.054	1.55	0.0395	0.276
		June	1	25	0.039	1.023	0.379	1
		August	1	27	0.043	1.20	0.134	0.939
		October	1	27	0.039	1.082	0.291	1
		December	1	28	0.043	1.24	0.0751	0.526
	Jaccard	Start	1	26	0.078	2.19	0.00230	0.0161
		February	1	25	0.057	1.52	0.00750	0.0525
		April	1	27	0.048	1.36	0.0310	0.217
		June	1	25	0.038	0.98	0.455	1
		August	1	27	0.04	1.13	0.126	0.885
		October	1	27	0.038	1.056	0.280	1
		December	1	28	0.039	1.13	0.0863	0.604

† P-values were corrected for multiple pairwise comparisons (n=7 sampling months) using a Bonferroni correction.

Table S7. Pairwise PERMANOVA across time of microbial communities extracted from *M. vimineum* litter translocated to the experimental site. PERMANOVA was conducted using the *pairwise.adonis2* function and 10,000 permutations. P-values obtained from *pairwise.adonis2* were corrected for multiple comparisons using a Bonferroni correction (n=21 comparisons). 0.002 is the lowest q-value obtainable with 10,000 permutations following a multiple comparison correction, thus q-values were labeled <0.002 if they met this threshold.

Infection	Distance	Months compared	DF	Residual DF	R^2	F-value	p-value	q-value
Healthy	Bray-Curtis	Start vs February	1	43	0.119	5.83	<0.001	<0.002
		Start vs April	1	45	0.270	16.62	<0.001	<0.002
		Start vs June	1	44	0.713	109.45	<0.001	<0.002
		Start vs August	1	45	0.806	186.54	<0.001	<0.002
		Start vs October	1	45	0.788	167.30	<0.001	<0.002
		Start vs December	1	46	0.800	183.81	<0.001	<0.002
		February vs April	1	44	0.119	5.97	0.00220	0.0462
		February vs June	1	43	0.642	77.23	<0.001	<0.002
		February vs August	1	44	0.767	144.50	<0.001	<0.002
		February vs October	1	44	0.746	128.97	<0.001	<0.002
		February vs December	1	45	0.758	140.58	<0.001	<0.002
		April vs June	1	45	0.375	26.96	<0.001	<0.002
		April vs August	1	46	0.565	59.82	<0.001	<0.002
		April vs October	1	46	0.540	53.96	<0.001	<0.002
		April vs December	1	47	0.545	56.28	<0.001	<0.002
		June vs August	1	45	0.204	11.52	<0.001	<0.002
		June vs October	1	45	0.178	9.77	<0.001	<0.002
		June vs December	1	46	0.163	8.94	<0.001	<0.002
		August vs October	1	46	0.019	0.90	0.405	1
		August vs December	1	47	0.017	0.82	0.509	1
		October vs December	1	47	0.012	0.57	0.744	1
	Jaccard	Start vs February	1	43	0.115	5.60	<0.001	0.00420
		Start vs April	1	45	0.268	16.49	<0.001	<0.002
		Start vs June	1	44	0.692	99.03	<0.001	<0.002
		Start vs August	1	45	0.778	157.31	<0.001	<0.002
		Start vs October	1	45	0.762	143.75	<0.001	<0.002
		Start vs December	1	46	0.771	154.93	<0.001	<0.002
		February vs April	1	44	0.114	5.64	0.00130	0.0273
		February vs June	1	43	0.617	69.21	<0.001	<0.002
		February vs August	1	44	0.733	120.92	<0.001	<0.002
		February vs October	1	44	0.714	109.67	<0.001	<0.002
		February vs December	1	45	0.723	117.68	<0.001	<0.002
		April vs June	1	45	0.359	25.17	<0.001	<0.002
		April vs August	1	46	0.530	51.97	<0.001	<0.002
		April vs October	1	46	0.507	47.36	<0.001	<0.002
		April vs December	1	47	0.511	49.17	<0.001	<0.002
		June vs August	1	45	0.179	9.82	<0.001	<0.002
		June vs October	1	45	0.159	8.54	<0.001	<0.002
		June vs December	1	46	0.143	7.68	<0.001	<0.002
		August vs October	1	46	0.019	0.88	0.438	1
		August vs December	1	47	0.017	0.81	0.542	1
		October vs December	1	47	0.013	0.63	0.733	1
Infected	Bray-Curtis	Start vs February	1	44	0.118	5.91	<0.001	<0.002
		Start vs April	1	44	0.298	18.71	<0.001	<0.002
		Start vs June	1	42	0.624	69.68	<0.001	<0.002
		Start vs August	1	44	0.780	155.87	<0.001	<0.002
		Start vs October	1	43	0.808	180.85	<0.001	<0.002
		Start vs December	1	44	0.763	141.97	<0.001	<0.002
		February vs April	1	46	0.206	11.96	<0.001	<0.002
		February vs June	1	44	0.603	66.80	<0.001	<0.002

	February vs August	1	46	0.772	155.74	<0.001	<0.002
	February vs October	1	45	0.791	170.59	<0.001	<0.002
	February vs December	1	46	0.756	142.76	<0.001	<0.002
	April vs June	1	44	0.337	22.40	<0.001	<0.002
	April vs August	1	46	0.632	79.16	<0.001	<0.002
	April vs October	1	45	0.639	79.55	<0.001	<0.002
	April vs December	1	46	0.607	70.93	<0.001	<0.002
	June vs August	1	44	0.291	18.05	<0.001	<0.002
	June vs October	1	43	0.271	16.00	<0.001	<0.002
	June vs December	1	44	0.255	15.08	<0.001	<0.002
	August vs October	1	45	0.043	2.02	0.0954	1
	August vs December	1	46	0.018	0.85	0.439	1
	October vs December	1	45	0.018	0.84	0.474	1
Jaccard	Start vs February	1	44	0.109	5.40	<0.001	<0.002
	Start vs April	1	44	0.283	17.34	<0.001	<0.002
	Start vs June	1	42	0.595	61.66	<0.001	<0.002
	Start vs August	1	44	0.740	125.12	<0.001	<0.002
	Start vs October	1	43	0.763	138.62	<0.001	<0.002
	Start vs December	1	44	0.722	114.31	<0.001	<0.002
	February vs April	1	46	0.200	11.49	<0.001	<0.002
	February vs June	1	44	0.578	60.35	<0.001	<0.002
	February vs August	1	46	0.736	127.91	<0.001	<0.002
	February vs October	1	45	0.752	136.10	<0.001	<0.002
	February vs December	1	46	0.719	117.59	<0.001	<0.002
	April vs June	1	44	0.319	20.59	<0.001	<0.002
	April vs August	1	46	0.591	66.42	<0.001	<0.002
	April vs October	1	45	0.594	65.82	<0.001	<0.002
	April vs December	1	46	0.564	59.44	<0.001	<0.002
	June vs August	1	44	0.260	15.47	<0.001	<0.002
	June vs October	1	43	0.239	13.49	<0.001	<0.002
	June vs December	1	44	0.224	12.71	<0.001	<0.002
	August vs October	1	45	0.046	2.16	0.0764	1
	August vs December	1	46	0.020	0.92	0.394	1
	October vs December	1	45	0.019	0.85	0.471	1

Table S8: Results of ANCOMBC2 testing the differential abundance (by infection status) of individual ASVs for each timepoint. Results are shown for ASVs which had a Holm-adjusted p-value (q-value) <0.05 and underwent a natural log fold change of at least 2. Each row (ASV x Month) represents a colored dot in Figure 4A. Negative natural log fold changes indicate an ASV was more prominent in infected litter. Functions were assigned through manual inspection of FUNGuild results and searches of the literature, ASVs which were not identified to at least the family level were functionally categorized as, "Unknown".

Month	Taxa	Function	% Reads	Natural log fold change (se)	W-statistic	p-value	q-value
Start	Periconia	Both	2.48	-3.44 (0.365)	-9.40	<0.001	<0.001
Start	Didymellaceae	Plant-associated	1.81	-2.27 (0.294)	-7.70	<0.001	<0.001
Start	Periconia	Both	1.68	-3.11 (0.290)	-10.71	<0.001	<0.001
Start	Ceramothyrium	Both	0.14	2.24 (0.201)	11.15	<0.001	<0.001
Start	Mycosphaerellaceae	Plant-associated	0.12	2.48 (0.168)	14.74	<0.001	<0.001
Start	Ceramothyrium	Both	0.08	2.21 (0.254)	8.72	<0.001	0.0113
Start	Ceramothyrium	Both	0.06	2.49 (0.227)	10.97	<0.001	0.00211
Start	Neoascochyta	Plant-associated	0.05	-5.05 (0.165)	-30.60	<0.001	0.00337
Start	Aureobasidium	Both	0.04	2.09 (0.197)	10.60	<0.001	<0.001
Start	Alfaria	Saprophytic	0.03	2.33 (0.227)	10.27	<0.001	<0.001
Start	Sporobolomyces	Saprophytic	0.02	2.33 (0.206)	11.30	<0.001	0.0138
Start	Bulleribasidium	Saprophytic	0.01	-2.34 (0.193)	-12.15	<0.001	<0.001
Start	Chaetothyriales	Unknown	0.01	3.46 (0.156)	22.17	<0.001	0.0118
February	Periconia	Both	1.07	-2.19 (0.315)	-6.96	<0.001	<0.001
February	Magnaporthaceae	Plant-associated	0.29	-2.17 (0.280)	-7.75	<0.001	0.00254
February	Pleosporales	Unknown	0.08	-3.69 (0.201)	-18.40	<0.001	0.0243
February	Phialophora	Saprophytic	0.08	2.43 (0.211)	11.49	<0.001	0.00411
February	Sordariomycetes	Unknown	0.05	-2.56 (0.273)	-9.38	<0.001	<0.001
February	Trichomeriaceae	Plant-associated	0.04	2.11 (0.208)	10.12	<0.001	0.00374
February	Keissleriella	Saprophytic	0.03	-2.11 (0.206)	-10.25	<0.001	<0.001
February	Neodevriesia	Saprophytic	0.03	2.08 (0.177)	11.74	<0.001	0.0369
February	Tripospermum	Saprophytic	0.02	2.28 (0.201)	11.35	<0.001	0.0134
February	Phaeosphaeria	Plant-associated	0.01	2.18 (0.183)	11.88	<0.001	0.0350
April	Apodus	Saprophytic	0.87	4.02 (0.217)	18.56	<0.001	0.00410
April	Flagellospora	Saprophytic	0.48	2.48 (0.277)	8.92	<0.001	0.00961
April	Helotiaceae	Saprophytic	0.37	3.11 (0.219)	14.18	<0.001	0.0152
April	Periconia	Both	0.36	-2.54 (0.334)	-7.62	<0.001	<0.001
April	Articulospora	Saprophytic	0.30	-2.57 (0.311)	-8.28	<0.001	<0.001
April	Spirosphaera	Saprophytic	0.24	2.73 (0.254)	10.72	<0.001	0.0188

April	Tetraplosphaeria	Plant-associated	0.12	-2.52 (0.252)	-10.01	<0.001	<0.001
April	Tetracladium	Saprophytic	0.10	2.13 (0.238)	8.94	<0.001	0.00443
April	Tetraplosphaeria	Plant-associated	0.09	-3.30 (0.233)	-14.13	<0.001	<0.001
April	Venturia	Plant-associated	0.05	2.39 (0.245)	9.76	<0.001	0.0322
June	Melanommataceae	Saprophytic	0.68	-2.41 (0.344)	-7.00	<0.001	0.00138
June	Melanommataceae	Saprophytic	0.44	-2.32 (0.296)	-7.85	<0.001	0.00210
June	Hyaloscyphaceae	Saprophytic	0.27	-4.56 (0.275)	-16.60	<0.001	0.00666
June	Melanommataceae	Saprophytic	0.05	2.12 (0.208)	10.19	<0.001	0.0237
June	Didymocyrtis	Other	0.05	2.21 (0.184)	11.99	<0.001	0.0323
June	Plectosphaerella	Saprophytic	0.03	3.18 (0.201)	15.83	<0.001	0.00839
June	Tetracladium	Saprophytic	0.02	2.19 (0.190)	11.49	<0.001	0.00391
August	Phallus	Saprophytic	0.64	3.96 (0.294)	13.48	<0.001	0.0171
August	Deconica	Saprophytic	0.30	4.30 (0.263)	16.35	<0.001	0.0340
August	Clitocybe	Saprophytic	0.26	-5.38 (0.174)	-30.92	<0.001	0.0309
August	Tubaria	Saprophytic	0.07	3.12 (0.205)	15.19	<0.001	0.0452
August	Tremellales	Unknown	0.06	-3.03 (0.177)	-17.09	<0.001	0.0286
August	Ceratocystis	Both	0.05	-2.37 (0.157)	-15.03	<0.001	0.0471
August	Tremellales	Unknown	0.04	-2.47 (0.189)	-13.07	<0.001	0.0198
August	Cercospora	Plant-associated	0.02	-2.65 (0.180)	-14.68	<0.001	0.00271
August	Phaeosphaeria	Plant-associated	0.01	2.19 (0.160)	13.68	<0.001	0.0159
October	Pleomassariaceae	Saprophytic	0.73	2.81 (0.296)	9.50	<0.001	0.0462
October	Helotiales	Unknown	0.66	-2.92 (0.261)	-11.20	<0.001	0.00619
October	Pleomassariaceae	Saprophytic	0.59	-2.71 (0.264)	-10.29	<0.001	0.00419
October	Pseudodactylaria	Saprophytic	0.51	2.18 (0.316)	6.91	<0.001	0.00988
October	Helotiales	Unknown	0.32	2.44 (0.205)	11.91	<0.001	0.0442
October	Phialocephala	Saprophytic	0.10	2.16 (0.178)	12.10	<0.001	0.00370
October	Deconica	Saprophytic	0.08	-2.18 (0.243)	-8.97	<0.001	0.00540
October	Helotiales	Unknown	0.04	-2.01 (0.188)	-10.70	<0.001	0.0239
October	Pleosporales	Unknown	0.03	2.12 (0.214)	9.90	<0.001	0.00240
October	Leotiomycetes	Unknown	0.02	2.05 (0.199)	10.30	<0.001	0.0296
October	Ascomycota	Unknown	0.01	-2.45 (0.197)	-12.46	<0.001	0.0355
October	Cylindrium	Saprophytic	0.01	2.38 (0.166)	14.32	<0.001	0.0182
December	Chloridium	Saprophytic	0.50	4.13 (0.243)	17.01	<0.001	0.0361
December	Pleosporales	Unknown	0.44	2.54 (0.382)	6.65	<0.001	0.0189
December	Chloridium	Saprophytic	0.36	2.79 (0.248)	11.27	<0.001	0.0489
December	Deconica	Saprophytic	0.36	2.53 (0.248)	10.17	<0.001	0.0101
December	Pleomassariaceae	Saprophytic	0.34	-2.02 (0.250)	-8.10	<0.001	0.0208
December	Cistella	Saprophytic	0.06	2.05 (0.204)	10.06	<0.001	0.0108
December	Trichomeriaceae	Plant-associated	0.03	-3.18 (0.196)	-16.23	<0.001	0.0432
December	Ascomycota	Unknown	0.02	2.83 (0.179)	15.83	<0.001	0.0476
December	Periconia	Both	0.02	2.10 (0.165)	12.73	<0.001	<0.001
December	Periconia	Both	0.02	3.16 (0.190)	16.65	<0.001	0.0391
December	Metacordyceps	Other	0.01	-2.03 (0.197)	-10.28	<0.001	0.00940

Table S9. ANOVA examining the impact of site and month of sampling on the overall, leaf, and stem mass remaining as well as the acid detergent lignin content and the Carbon:Nitrogen ratio for comparison between experimental site litter versus translocated litter. Transformations used in each analysis are indicated in the table.

		Transformation		NumDF	DenDF	F-value	p-value
Mass Remaining	Overall	None	Site†	1	4.31	25.56	0.00589
			Month	5	157.28	45.17	<0.001
			Site:Month	5	157.28	1.11	0.357
	Leaf	None	Site	1	4.04	4.20	0.109
			Month	3	101.01	21.84	<0.001
			Site:Month	3	101.04	1.59	0.196
	Stem	Squared	Site	1	4.03	0.15	0.716
			Month	3	101.12	25.75	<0.001
			Site:Month	3	101.14	0.29	0.832
Lignin		Square Root	Site	1	4.03	3.54	0.132
			Month	6	183.12	9.05	<0.001
			Site:Month	6	183.12	1.49	0.185
C:N Ratio		Log	Site	1	3.99	2.50	0.189
			Month	6	183.06	40.69	<0.001
			Site:Month	6	183.06	2.17	0.0482

†Site - local (from experimental site) vs translocated litter source, all healthy.

Table S10. Results of betadisper, multivariate test for homology of variance in dissimilarity distances. Distances matrices were formed using Bray-Curtis and Jaccard distances metrics.

Litter Source	Month	Bray-Curtis	Jaccard
Healthy	Start	0.3746	0.4817
	February	0.4481	0.5415
	April	0.5140	0.5866
	June	0.5946	0.6362
	August	0.6340	0.6601
	October	0.6189	0.6514
	December	0.6378	0.6630
Infected	Start	0.4266	0.5230
	February	0.4390	0.5349
	April	0.5222	0.5935
	June	0.6032	0.6428
	August	0.6038	0.6396
	October	0.6187	0.6519
	December	0.6239	0.6530

Figure S1. Climate measurements during decomposition experiment. (Top) Seven-day average of daily highs (red) and lows (blue). (Bottom) Seven-day average of humidity (line) and precipitation (bars). Vertical dashed lines indicate collection dates.

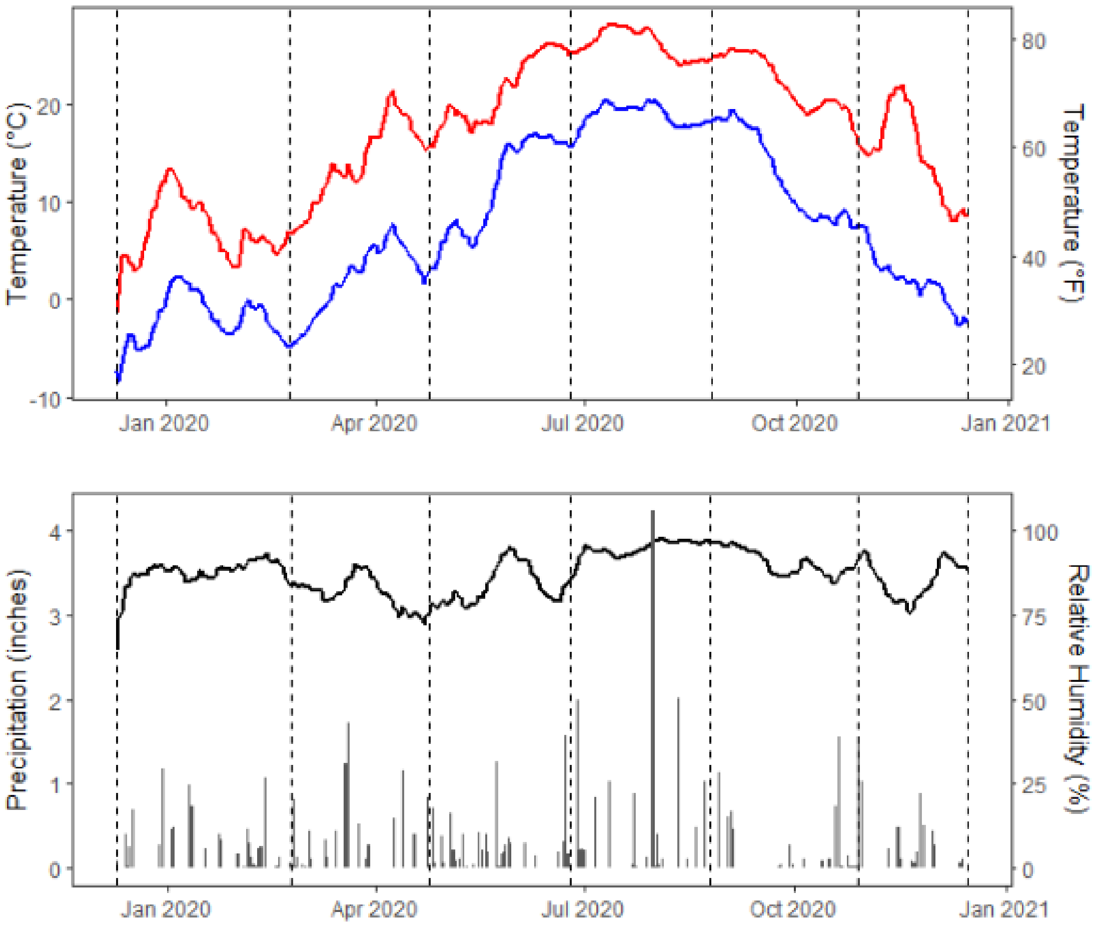

Figure S2. Percent of A) Overall Mass, B) Leaf Mass, C) Stem Mass, remaining at each timepoint. Overall tissue and leaf tissue, but not stem tissue, originating from the common garden site was significantly more decomposed than translocated healthy tissue. Data points from litter originating from the common garden experimental site are offset to the right of each timepoint while points from healthy litter sites are offset to the left. Shaded region represents 95% CI.

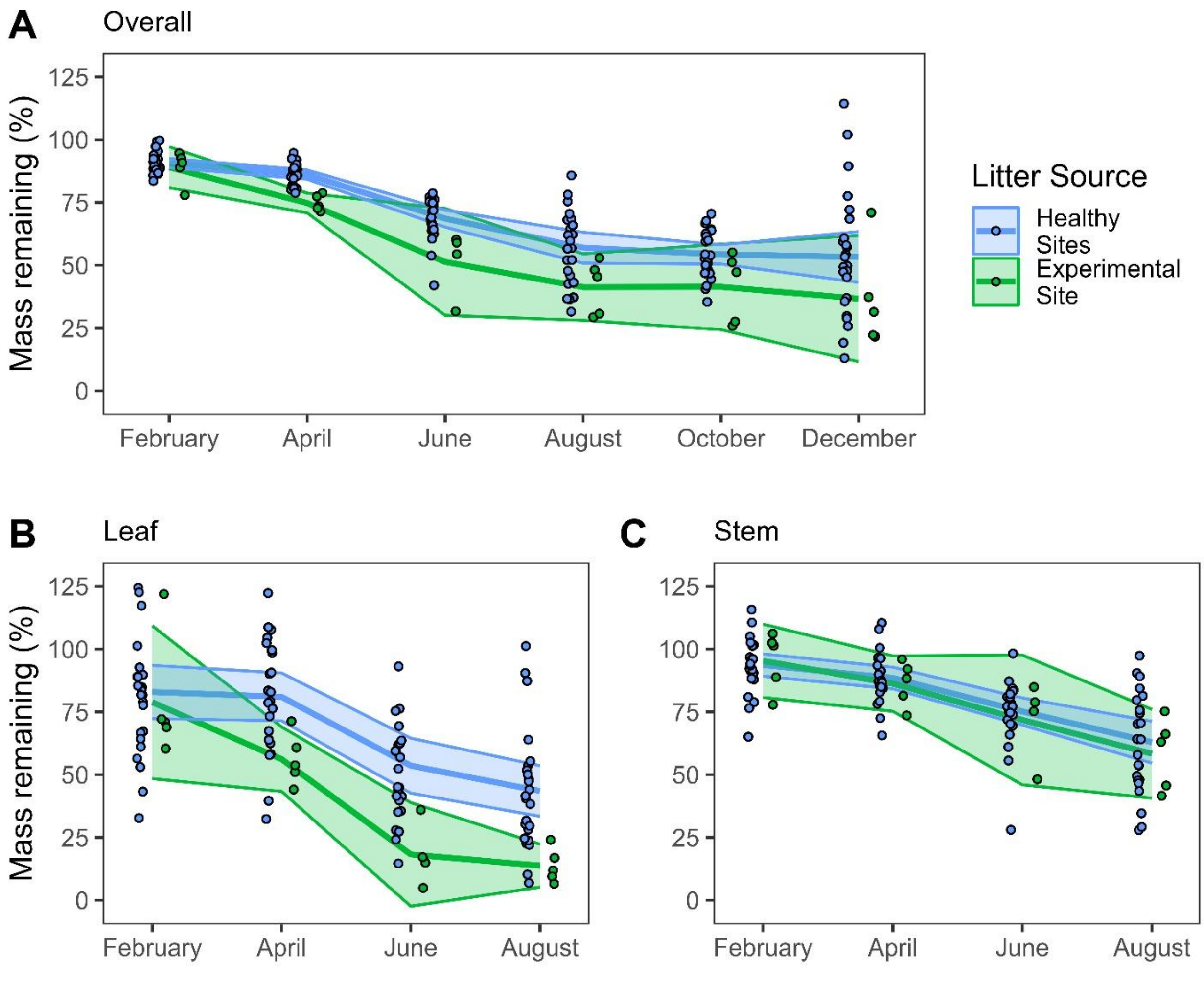

Supporting references:

Bolyen, E., J. R. Rideout, M. R. Dillon, N. A. Bokulich, C. C. Abnet, G. A. Al-Ghalith, H. Alexander, E. J. Alm, M. Arumugam, F. Asnicar, Y. Bai, J. E. Bisanz, K. Bittinger, A. Brejnrod, C. J. Brislawn, C. T. Brown, B. J. Callahan, A. M. Caraballo-Rodríguez, J. Chase, E. K. Cope, R. Da Silva, C. Diener, P. C. Dorrestein, G. M. Douglas, D. M. Durall, C. Duvallet, C. F. Edwardson, M. Ernst, M. Estaki, J. Fouquier, J. M. Gauglitz, S. M. Gibbons, D. L. Gibson, A. Gonzalez, K. Gorlick, J. Guo, B. Hillmann, S. Holmes, H. Holste, C. Huttenhower, G. A. Huttley, S. Janssen, A. K. Jarmusch, L. Jiang, B. D. Kaehler, K. B. Kang, C. R. Keefe, P. Keim, S. T. Kelley, D. Knights, I. Koester, T. Kosciolek, J. Kreps, M. G. I. Langille, J. Lee, R. Ley, Y.-X. Liu, E. Loftfield, C. Lozupone, M. Maher, C. Marotz, B. D. Martin, D. McDonald, L. J. McIver, A. V. Melnik, J. L. Metcalf, S. C. Morgan, J. T. Morton, A. T. Naimey, J. A. Navas-Molina, L. F. Nothias, S. B. Orchanian, T. Pearson, S. L. Peoples, D. Petras, M. L. Preuss, E. Pruesse, L. B. Rasmussen, A. Rivers, M. S. Robeson, P. Rosenthal, N. Segata, M. Shaffer, A. Shiffer, R. Sinha, S. J. Song, J. R. Spear, A. D. Swafford, L. R. Thompson, P. J. Torres, P. Trinh, A. Tripathi, P. J. Turnbaugh, S. Ul-Hasan, J. J. J. van der Hooft, F. Vargas, Y. Vázquez-Baeza, E. Vogtmann, M. von Hippel, W. Walters, Y. Wan, M. Wang, J. Warren, K. C. Weber, C. H. D. Williamson, A. D. Willis, Z. Z. Xu, J. R. Zaneveld, Y. Zhang, Q. Zhu, R. Knight, and J. G. Caporaso. 2019. Reproducible, interactive, scalable and extensible microbiome data science using QIIME 2. Nature Biotechnology 37:852–857.

Brazee, N. J., and D. L. Lindner. 2013. Unravelling the *P hellinus pini* s.l. complex in N orth A merica: a multilocus phylogeny and differentiation analysis of *P orodaedalea* . Forest Pathology 43:132–143.

Callahan, B. J., P. J. McMurdie, M. J. Rosen, A. W. Han, A. J. A. Johnson, and S. P. Holmes. 2016. DADA2: High-resolution sample inference from Illumina amplicon data. Nature Methods 13:581–583.

Davis, N. M., D. M. Proctor, S. P. Holmes, D. A. Relman, and B. J. Callahan. 2018. Simple statistical identification and removal of contaminant sequences in marker-gene and metagenomics data. Microbiome 6:226.

Ihrmark, K., I. T. M. Bödeker, K. Cruz-Martinez, H. Friberg, A. Kubartova, J. Schenck, Y. Strid, J. Stenlid, M. Brandström-Durling, K. E. Clemmensen, and B. D. Lindahl. 2012. New primers to amplify the fungal ITS2 region - evaluation by 454-sequencing of artificial and natural communities. FEMS Microbiology Ecology 82:666–677.

Lane, B. R., A. E. Kendig, C. M. Wojan, A. Adhikari, M. A. Jusino, N. Kortessis, M. W. Simon, R. D. Holt, M. E. Smith, K. Clay, S. L. Flory, P. F. Harmon, and E. M. Goss. 2023. Fungicide-Mediated Shifts in the Foliar Fungal Community of an Invasive Grass. Phytobiomes Journal 7:198–207.

McMurdie, P. J., and S. Holmes. 2013. phyloseq: An R Package for Reproducible Interactive Analysis and Graphics of Microbiome Census Data. PLoS ONE 8:e61217.

Nilsson, R. H., K.-H. Larsson, A. F. S. Taylor, J. Bengtsson-Palme, T. S. Jeppesen, D. Schigel, P. Kennedy, K. Picard, F. O. Glöckner, L. Tedersoo, I. Saar, U. Kõljalg, and K. Abarenkov. 2019. The UNITE database for molecular identification of fungi: handling dark taxa and parallel taxonomic classifications. Nucleic Acids Research 47:D259–D264.

Oksanen, J., G. L. Simpson, F. G. Blanchet, R. Kindt, P. Legendre, P. R. Minchin, R. B. O’Hara, P. Solymos, M. H. H. Stevens, E. Szoecs, H. Wagner, M. Barbour, M. Bedward, B. Bolker, D. Borcard, G. Carvalho, M. Chirico, M. D. Caceres, S. Durand, H. B. A. Evangelista, R.

FitzJohn, M. Friendly, B. Furneaux, G. Hannigan, M. O. Hill, L. Lahti, D. McGlinn, M.-H. Ouellette, E. R. Cunha, T. Smith, A. Stier, C. J. F. T. Braak, and J. Weedon. 2022. vegan: Community Ecology Package.

White, T. J., T. Bruns, S. Lee, and J. Taylor. 1990. Amplification and direct sequencing of fungal ribosomal RNA genes for phylogenetics. Pages 315–322 PCR Protocols. Elsevier.
